# Attenuation of acrylamide-induced neurotoxicity by supplementation of sitagliptin in Wistar rats

**DOI:** 10.22038/IJBMS.2023.73187.15905

**Published:** 2024

**Authors:** Mahboobeh Navabi, Mahboobeh Ghasemzadeh Rahbardar, Soghra Mehri, Hossein Hosseinzadeh

**Affiliations:** 1 Department of Pharmacodynamics and Toxicology, School of Pharmacy, Mashhad University of Medical Sciences, Mashhad, Iran; 2 Pharmaceutical Research Center, Pharmaceutical Technology Institute, Mashhad University of Medical Sciences, Mashhad, Iran

**Keywords:** Anti-oxidants, Caspases, Glutathione, Inflammation, Malondialdehyde, Neurotoxicity syndromes, Tumor necrosis factors

## Abstract

**Objective(s):**

Acrylamide (ACR) induces neurotoxicity in humans and animals through different mechanisms. Sitagliptin is a type-2 diabetes medication with neuroprotective properties. The effects of sitagliptin against neurotoxicity stimulated by ACR were examined.

**Materials and Methods:**

Male Wistar rats were classified as follows: 1. Control (normal saline, 11 days, IP), 2. ACR (50 mg/kg, 11 days, IP), 3. ACR (11 days, days 11-20 normal saline), 4-7. ACR+sitagliptin (5, 10, 20, and 40 mg/kg, 11 days, IP), 8. ACR+sitagliptin (10 mg/kg, days 6-11), 9. ACR+sitagliptin (10 mg/kg, days 6-20), 10. Sitagliptin (40 mg/kg, 11 days), 11. ACR+vitamin E (200 mg/kg, IP). Finally, the gait score was evaluated. Reduced glutathione (GSH) and malondialdehyde (MDA) levels were measured in cortex tissue. Also, IL-1β, TNF-α, and caspase-3 levels were assessed in the cortex by western blotting.

**Results:**

ACR caused movement disorders, triggered oxidative stress, and raised TNF-α, IL-1β, and caspase-3 cleaved levels. Supplementation of sitagliptin (10 mg/kg) along with ACR, in 3 protocols, reduced gait disorders compared to the ACR group. Receiving sitagliptin in all doses plus ACR and injection of sitagliptin (10 mg/kg) from days 6 to11 reduced the MDA level of cortex tissue. Sitagliptin (all doses) plus ACR increased the GSH level of the cortex tissue. Sitagliptin (10 mg/kg) with ACR dropped the amounts of TNF-α and caspase-3 cleaved proteins in cortex tissue but did not affect the IL-1β level.

**Conclusion:**

Sitagliptin disclosed preventive and therapeutic effects on ACR neurotoxicity. Sitagliptin possesses antioxidant, anti-inflammatory, and anti-apoptotic properties and inhibits CR neurotoxicity in rats.

## Introduction

Acrylamide (ACR) is generated throughout the cooking of starchy products at temperatures above 120 °C due to the Maillard reaction. It has been found in a variety of baked and fried carbohydrate-rich foods, including, breakfast cereals, bread, crisps, coffee, and potato chips (1, 2). Adults consume approximately 0.5 µg/kg/bw of ACR daily (2). The common uptake of ACR for adults might range from 14 to 70 µg/day based upon a typical body weight of 70 kg, while for children it might vary from 70 to 280 µg/day. Recent studies have shown that ACR causes cytotoxicity (3), neurotoxicity (4, 5), carcinogenicity (1), hepatotoxicity (6, 7), and metabolic syndrome (8) which can cause a severe burden on individuals (9, 10). 

Neuropathies, ataxia, cerebellar alterations (in rare cases), numbness in the feet and hands, gait irregularities, muscle weakness, and skin changes, are all symptoms of sub-chronic exposure to this toxin (11). Furthermore, ACR leads to neuronal loss in central and peripheral nervous systems (4). Neurotoxicity resulting from ACR exposure can be attributed to multiple mechanisms. One of the primary drivers involves the generation of oxidative stress. Additionally, ACR has been shown to trigger apoptosis (2) and inflammation (12). ACR administration dramatically lowers brain reduced glutathione (GSH) levels, superoxide dismutase, glutathione peroxidase, and catalase function. It also raises malondialdehyde (MDA), interleukin-1β (IL-1β), nitric oxide (NO), and tumor necrosis factor-α (TNF-α) amounts (13). In another study, ACR caused GSH depletion in rat hepatocytes (14). Another study has shown that ACR increased the ratio of Bcl-2-associated X protein (Bax)/B-cell lymphoma 2 (Bcl-2) and caspase-3 cleaved, causing severe movement disorders (2). As a result, ACR-induced neurotoxicity appears to necessitate the use of effective medications with fewer side effects.

Sitagliptin is a medication utilized to amend type 2 diabetes mellitus. It belongs to the dipeptidyl peptidase-4 inhibitor group (15) and increases insulin production and decreases glucagon secretion (16). Anti-oxidant (17), anti-inflammatory (17, 18), and anti-apoptotic (16) properties are essential mechanisms of this drug in creating neuroprotective effects. Furthermore, studies showed that TNF-𝛼, IL-6, and IL-17 levels were reduced by sitagliptin, whereas anti-inflammatory markers, for instance, transforming growth factor-β (TGF-β) and IL-10 amounts were increased (16). El Sahar *et al.* revealed that sitagliptin injection lowered glutamate and NO levels while increased GSH concentration inside hippocampal structures in rats (19). One study showed that sitagliptin reduced caspase-3 and Bax levels, resulting in reduced renal apoptosis (20).

Based on the information provided, our team decided to test the therapeutic and protective effects of various doses of sitagliptin (5, 10, 20, and 40 mg/kg, IP) as a neuroprotective agent on ACR neurotoxicity. At the end of the treatment, gait score, MDA, GSH, IL-1β, TNF-α, and caspase-3 amounts were measured in rats’ cortex tissue. 

## Materials and Methods

***Chemicals***

The used materials are as follows: Sitagliptin (Tinab Shimi, Iran); polyvinylidene fluoride (PVDF) membrane (BioRad, USA); [ACR (C_3_H_5_NO, > 99% purity), thiobarbituric acid (TBA.), phosphoric acid, 5, 5′-Dithiobis 2-nitrobenzoic acid (DTNB), and potassium chloride (KCl) (Merck Company, Germany)]; Vitamin E (Tehran Darou Pharmaceutical Co., Tehran, Iran).

***Animals***

Wistar rats (male) weighing between 220 and 250 g were sourced from the animal quarters of the School of Pharmacy and housed in a controlled environment at a temperature of 22–25 °C. They were subjected to a 12-hour alternating light-dark cycle. There were no restrictions on the amount of water or food that could be consumed. The Mashhad University of Medical Sciences Animal Care and Use Committee granted authorization for all experiments, which were conducted in accordance with the internationally accepted principles for animal use and care. The study was also conducted in compliance with the IR.MUMS.PHARMACY.REC.1398.017 protocol.

***Induction of neurotoxicity***

To induce toxicity in male rats, a method of intraperitoneal injection was employed, where they were administered 50 mg/kg of ACR daily for 11 days (21, 22). To see if sitagliptin could protect against ACR-induced neurotoxicity, doses of 5, 10, 20, and 40 mg/kg were administered intraperitoneally for 11 days, and 30 min before receiving ACR. All injections were administered daily at the times specified. The animals in this investigation were divided into the following groups at random, with seven rats per group:

Group 1: Male rats receiving normal saline as the vehicle in the control group;

Group 2: Male rats receiving ACR (50 mg/kg, 11 days, IP); 

Group 3: Male rats receiving ACR (50 mg/kg, 11 days, IP) and received normal saline on days 11-20;

Group 4: Male rats receiving ACR (50 mg/kg, 11 days, IP)+sitagliptin (5 mg/kg, 11 days, IP);

Group 5: Male rats receiving ACR (50 mg/kg, 11 days, IP)+sitagliptin (10 mg/kg, 11 days, IP) (23);

Group 6: Male rats receiving ACR (50 mg/kg, 11 days, IP)+sitagliptin (20 mg/kg, 11 days, IP);

Group 7: Male rats receiving ACR (50 mg/kg, 11 days, IP)+sitagliptin (40 mg/kg, 11 days, IP); 

Group 8: Male rats receiving ACR (50 mg/kg, 11 days, IP)+sitagliptin (10 mg/kg, days 6-11, IP);

Group 9: Male rats receiving ACR (50 mg/kg, 11 days, IP)+sitagliptin (10 mg/kg, days 6-20, IP);

Group 10: Male rats receiving sitagliptin (40 mg/kg, 11 days, IP);

Group 11: Positive control group male rats receiving ACR (50 mg/kg, 11 days, IP)+ Vitamin E (200 mg/kg, every other day, IP) (2, 24).

***Assessment of the gait score***

Animals were positioned in a clear box and their movements were checked for three minutes at the end of the treatment period. The following is a ranking of their movements:

1: Normal walking.

2: Slightly affected steps and walking (minor weakness of the lower extremity). 

3: Moderately impaired steps and walking (moderate weakness of the lower extremity). 

4: Severe impact on steps and walking due to lower limb paralysis (25).

***Sample collection***

Animals were sacrificed after the gait analysis and the brains (cerebral cortex) were immediately collected and frozen using liquid nitrogen. The frozen brain tissues were then kept at -80 °C. Additionally, serum samples were obtained and stored at -80 °C until biochemical analysis.

***Assessing brain tissue GSH levels***

The GSH content in the tissues was determined using the method designated by Moron *et al*. with minor modifications (26). A 10% tissue homogenate was made using a buffer solution at pH 7.4. The homogenate was then combined with 10% trichloroacetic acid (TCA) and centrifuged for 10 min (3000 g). Next, 2.5 ml of phosphate buffer at pH 8 and DTNB were mixed with 0.5 ml of the resulting supernatant. The absorbance of the samples was measured at 412 nm using a Jenway 6105 UV/Vis spectrophotometer (UK). The GSH amounts were quantified in nmol/g tissue (27, 28).

***Assessing brain tissue MDA levels***

MDA is a lipid peroxidation marker that combines with thiobarbituric acid (TBA) to create a pink color complex with maximum absorption at the wavelength of 532 nm (29, 30). The 10% homogenate in 1.15% KCl was made to assess the amounts of MDA in isolated tissue. After that, 0.5 ml of homogenized tissue was combined with 3 ml of 1% phosphoric acid and 1 ml of 6% TBA. The tubes were then placed in boiling water, removed after 45 min, and cooled slowly. When the liquid had cooled, 4 ml of n-butanol was combined and blended for 1 min. The blend was centrifuged for 20 min (3000 g). Finally, the organic layer absorbance was measured at 532 nm with spectrophotometer (Jenway 6105 UV/Vis, UK). MDA was measured in nmol/g tissue (31, 32).

***Analysis of Western blots***

The tissue was homogenized in a lysis solution comprising 10 mM NaF, 2 mM ethylenediaminetetraacetic acid (EDTA), 50 mM Tris-HCl (pH=7.4), 2 mM ethylene glycol tetraacetic acid (EGTA), 1 mM sodium orthovanadate (Na_3_VO_4_), 0.2% W/V sodium deoxycholate, 10 mM glycerophosphate, 1 mM phenylmethylsulfonyl fluoride (Sigma Aldrich, USA). The Bradford assay kit (Bio-Rad, USA) was utilized to calculate the protein concentration in the samples. The samples were mixed with 2X sodium dodecyl sulfate (SDS) blue buffer and then boiled for 5 min before being aliquoted and kept at -80 °C (33).

The samples were subjected to electrophoresis on SDS polyacrylamide gels with a concentration of 12% (for IL-1β and TNF-α) or 15% (for caspase-3). Subsequently, the proteins were transferred from the gel onto a polyvinylidene fluoride (PVDF) membrane to facilitate protein detection. The PVDF membrane was placed on a rocker at room temperature and blocked with 5% skimmed milk in Tris-buffered saline tween 20 (TBST) for two hours. After that, the blots were washed three times with TBST and incubated for two hours at room temperature with rabbit polyclonal antiserum against TNF-a (Cell Signaling #3707, 1: 1,000, rabbit monoclonal anti-serum against IL-1β (Abcam #9722, 1: 1,000), rabbit monoclonal anti-serum against caspase-3 (Cell Signaling #9665, 1: 1000), and mouse monoclonal anti-serum against beta-actin (Cell Signaling, #3700, 1: 1,000). After three washes with TBST, the blots were incubated for 1.5 hr at room temperature with anti-rabbit IgG labeled with horseradish peroxidase (Cell Signaling, #7074, 1:3000) or anti-mouse IgG labeled with horseradish peroxidase (Cell Signaling, #7076, 1:3000).

The peroxidase-coated bands on the PVDF membrane were identified using an enhanced chemiluminescence system (Pierce, USA). The optical densities of the bands were quantified with the Alliance 4.7 Gel doc system (UK), and densitometric analysis of the protein bands was done by UVtec software (UK). To standardize the protein amounts, the intensities of the bands were normalized against the corresponding bands of β-actin, which served as a control protein.

***Statistical analysis***

For statistical analysis, GraphPad Prism 8.0 (GraphPad Prism Software Inc., San Diego, CA, USA) was utilized. In the lipid peroxidation experiment, GSH content test, and western blot analysis, the results were presented as Mean±SD. In the tests stated, statistical comparisons were done using one-way ANOVA followed by the Tukey–Kramer test. Statistical significance was defined as *P*<0.05, for the behavioral tests (gait abnormalities), data were described as medians with interquartile ranges, and statistical analysis was performed using the Kruskal–Wallis nonparametric test and Dunn’s Multiple Comparisons. The Mann-Whitney test was used to compare the two groups.

## Results

***Effect of sitagliptin on ACR-induced gait abnormalities***

The injection of ACR (50 mg/kg) for 11 days caused gait disturbance in animals compared to the control animals (*P*<0.0001). Sitagliptin (10 mg/kg) plus ACR for 11 days significantly reduced these disorders versus the ACR group (*P*<0.05), while receiving other doses of sitagliptin (5, 20, and 40 mg/kg) with ACR caused no significant difference versus the ACR group. Administration of sitagliptin (10 mg/kg) from day 6 to 11 along with ACR also made a substantial difference compared to the group receiving ACR (*P*<0.05). Besides, receiving vitamin E (200 mg/kg) and ACR significantly reduced the gait abnormality versus the ACR group (*P*<0.01). Supplementation of sitagliptin (40 mg/kg) for 11 days caused no remarkable alteration versus the control group.

**Figure 1:**
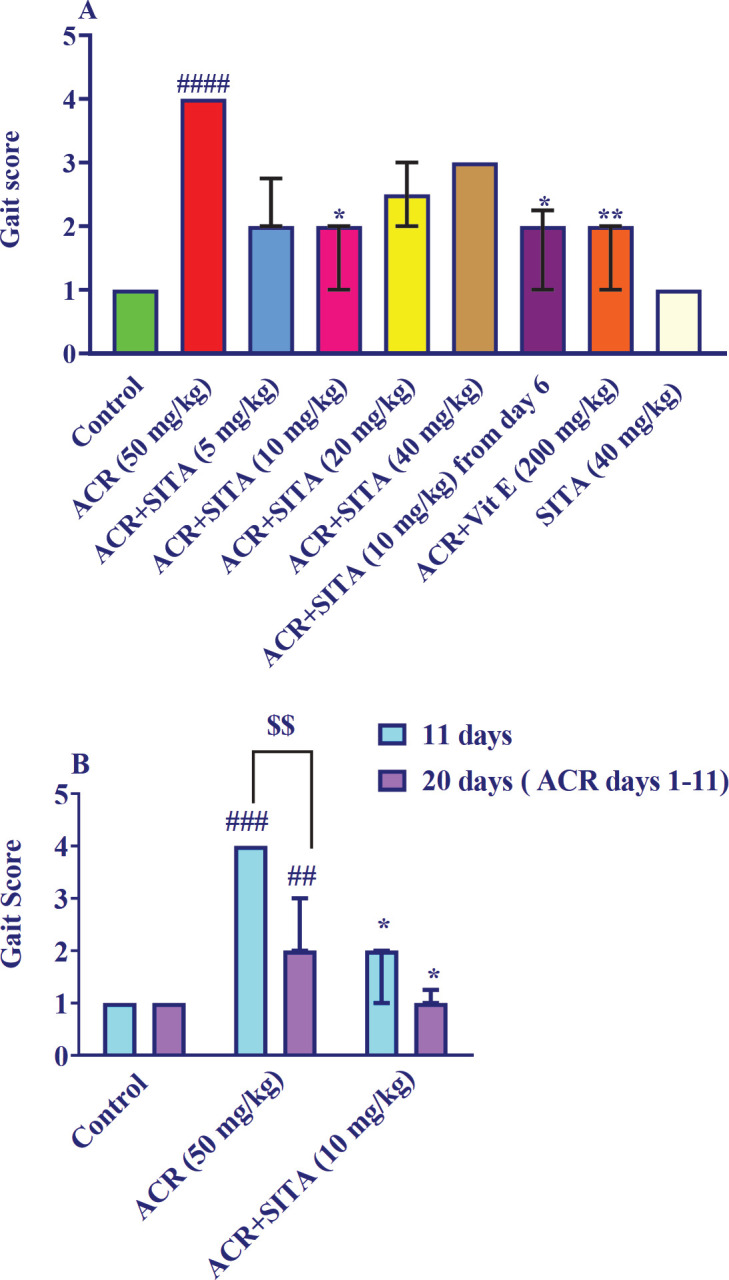
Effect of sitagliptin on ACR-induced gait abnormalities in Wistar rats

Figure 1B was drawn to answer the questions of whether animals would recover if ACR was discontinued and what the results would be if sitagliptin, which started on day 6, would continue until day 20. In the group that received ACR for 11 days and then the administration of ACR was stopped and up to day 20, the animals received only normal saline, compared to the group receiving ACR from days 1 to 11 significant difference was observed (*P*<0.01). Sitagliptin (10 mg/kg) plus ACR made a weighty difference (*P*<0.05) versus the ACR group (for 11 days). Likewise, in the group that received ACR from the beginning (day 1), sitagliptin (10 mg/kg) administration was started from day 6 and continued until day 20, compared to the group that received ACR for up to 11 days and was studied for up to 20 days, significant alteration was observed (*P*<0.05). Administration of sitagliptin up to day 20 did not significantly reduce ACR-induced disorders compared to administration up to day 11.

***Effect of sitagliptin on ACR-induced GSH changes in cortical tissue***

**Figure 2:**
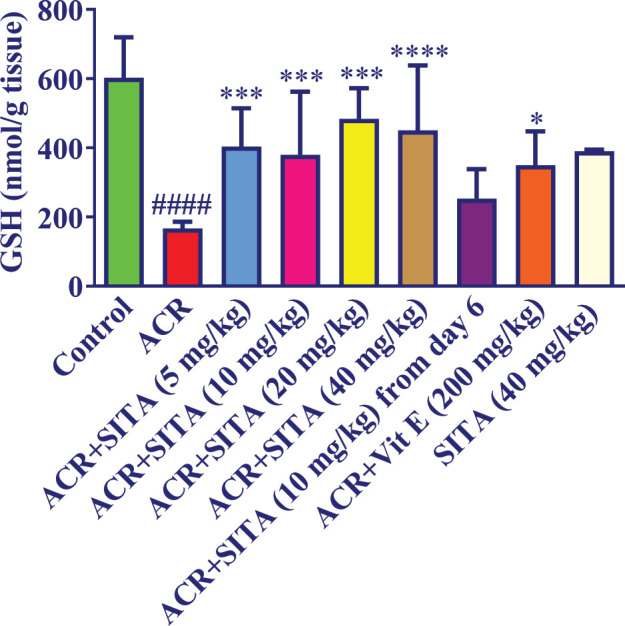
Effect of sitagliptin on ACR-induced lipid peroxidation in cortical tissue in Wistar rats

Injecting ACR (50 mg/kg) for 11 days significantly reduced the GSH content of the cortex compared to the control group (*P*<0.0001) (Figure 2). Co-administration of sitagliptin (5, 10, 20 mg/kg) (*P*<0.001) and 40 mg/kg (*P*<0.0001) and ACR augmented GSH levels versus the ACR group. Injection of sitagliptin (10 mg/kg) from day 6 in conjunction with ACR did not show a significant change in GSH level versus the ACR group. No remarkable difference was observed in the group receiving 40 mg/kg of sitagliptin alone compared to the control group. Moreover, concurrent supplementation of vitamin E (200 mg/kg) and ACR triggered a substantial increase in GSH content compared to the ACR group (*P*<0.05).

***Effect of sitagliptin on ACR-induced lipid peroxidation in cortical tissue***

**Figure 3:**
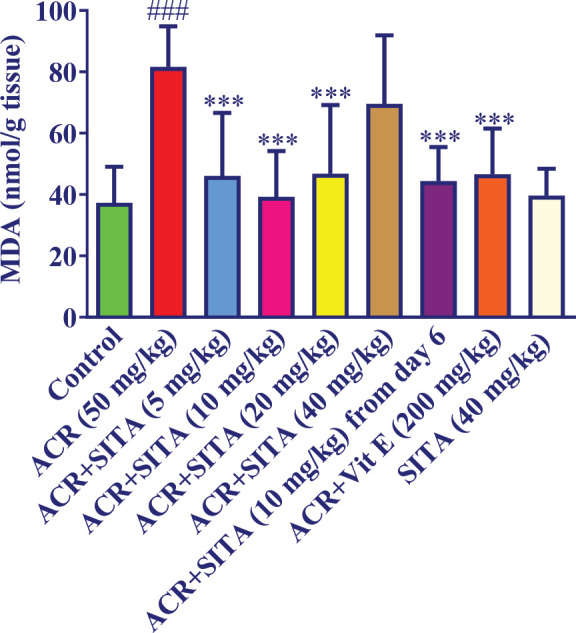
Effect of sitagliptin on ACR-induced lipid peroxidation in cortical tissue in Wistar rats

The results showed that administration of ACR (50 mg/kg) for 11 days triggered a substantial increase in the MDA level of cortical tissue versus the control rats (*P*<0.001). Administration of sitagliptin (5, 10, and 20 mg/kg) plus ACR for 11 days pointedly reduced the MDA level of cortical tissue versus the ACR group (*P*<0.001). However, receiving sitagliptin (40 mg/kg) along with ACR for 11 days did not alter the MDA level of cortical tissue compared to the ACR group. Furthermore, supplementation of sitagliptin (10 mg/kg) from day 6 plus ACR resulted in a substantial reduction in MDA level versus the ACR group (*P*<0.001). Administration of sitagliptin alone showed a non-significant alteration in MDA levels in comparison to the control group. Administration of vitamin E (200 mg/kg) with ACR was able to considerably reduce the level of MDA in cortical tissue versus the group receiving ACR (*P*<0.001) (Figure 3). 

***Effect of ACR and sitagliptin on the level of TNF-α in cortical tissue***

**Figure 4:**
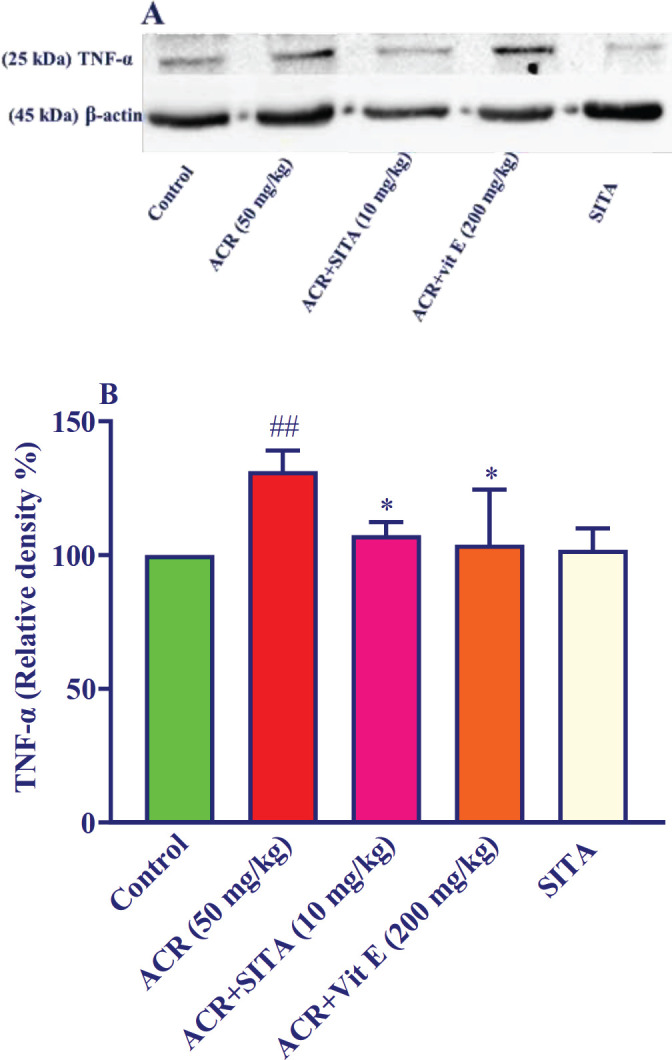
Effect of ACR and sitagliptin on the level of TNF-α in cortical tissue in Wistar rats

ACR (50 mg/kg) intraperitoneal injection for 11 days pointedly augmented the level of TNF-α protein in cortical tissue versus the control group (*P*<0.01). Co-administration of sitagliptin (10 mg/kg) and ACR triggered a noteworthy reduction in the level of TNF-α protein in cortical tissue versus the ACR group (*P*<0.05). Administration of sitagliptin alone did not show a substantial change in TNF-α amounts in cortical tissue in comparison to the control group. Vitamin E (200 mg/kg) plus ACR was able to significantly reduce the level of TNF-α protein in the cortex versus the group receiving ACR (*P*<0.05) (Figure 4). 

***Effect of ACR and sitagliptin on the level of IL-1β in cortical tissue***

**Figure 5:**
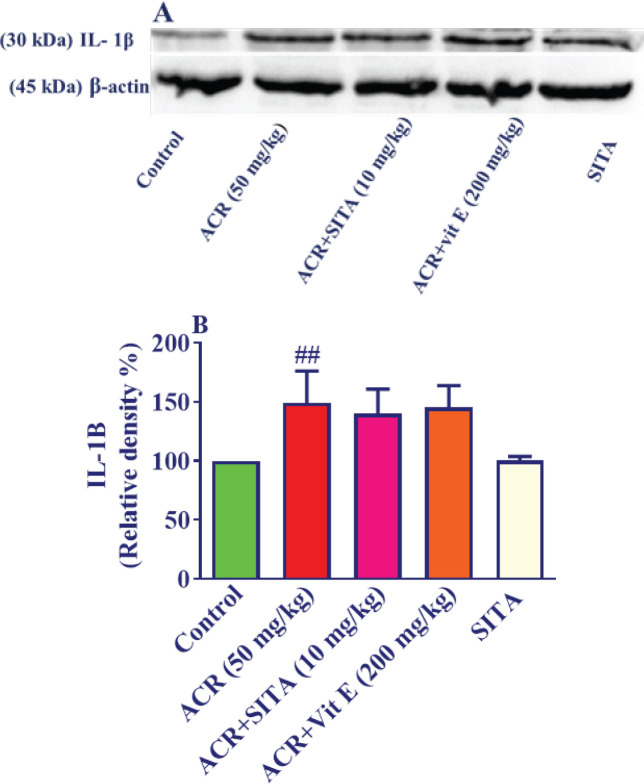
Effect of ACR and sitagliptin on the level of IL-1β in cortical tissue in Wistar rats

Intraperitoneal injection of ACR (50 mg/kg) for 11 days led to a noteworthy rise in IL-1β protein amount in cortical tissue versus the control group (*P*<0.01). Injecting sitagliptin (10 mg/kg) along with ACR for 11 days did not alter IL-1β amounts in cortical tissue versus the ACR group. Administration of sitagliptin alone did not pointedly affect IL-1β amounts in cortical tissue compared to control samples. Supplementation of vitamin E (200 mg/kg) and ACR could not reduce IL-1β amounts versus the ACR group (Figure 5).

***Effect of ACR and sitagliptin on the level of caspase-3 (pro & cleaved) in cortical tissue***

**Figure 6:**
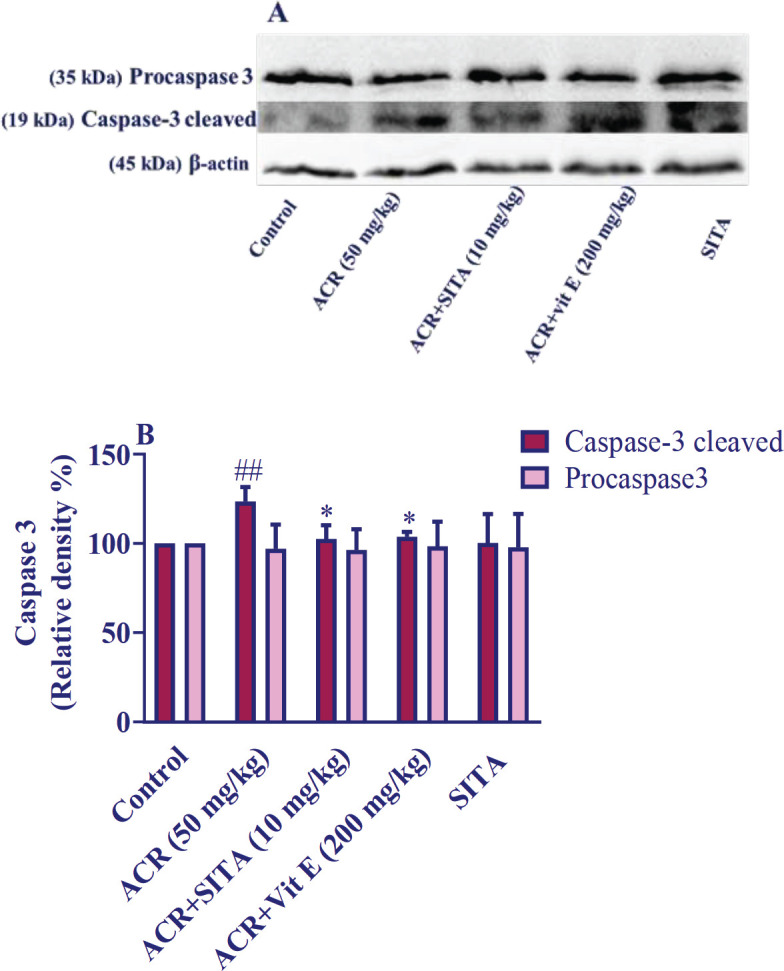
Effect of ACR and sitagliptin on the level of caspase-3 (pro & cleaved) in cortical tissue in Wistar rats

As shown in Figure 6, ACR (50 mg/kg) gave rise to a noteworthy upsurge in the level of caspase-3 cleaved protein in cortical tissue versus the control samples (*P*<0.01). Moreover, sitagliptin at a dose of 10 mg/kg plus ACR significantly lowered the amounts of caspase-3 cleaved protein versus the group receiving ACR (*P*<0.05). Prescription of sitagliptin alone did not alter the level of this protein in cortical tissue in comparison to controls. Co-administration of vitamin E (200 mg/kg) and ACR significantly decreased the level of caspase-3 cleaved protein versus the ACR group (*P*<0.05). No substantial alterations were disclosed in procaspase-3 protein levels in different groups following the administration of ACR and sitagliptin.

A: ACR (50 mg/kg), sitagliptin (5, 10, 20, and 40 mg/kg), and vitamin E (200 mg/kg) were injected intraperitoneally into the animals. The behavioral index was assessed after 11 days of administration. B: ACR (50 mg/kg) and sitagliptin (10 mg/kg) were injected intraperitoneally into the animals. The behavioral index was measured after 11 and 20 days. In both graphs A and B, the data are median with an interquartile range (n=7). Kruskal-Wallis non-parametric tests, Dunn's multiple comparisons test, and Mann-Whitney test were used to examine the statistical differences. #### *P*<0.0001, ### *P*<0.001, ## *P*<0.01 compared to the control group, ** *P*<0.01, * *P*<0.05 compared to the ACR group and $$ P<0.01 compared groups on days 11 and 20.

ACR: Acrylamide; Sita: Sitagliptin; Vit E: Vitamin E

ACR (50 mg/kg), sitagliptin (5, 10, 20, and 40 mg/kg), and vitamin E (200 mg/kg) were injected intraperitoneally to the animals. The data is Mean±SD (n=7). ANOVA test and Tukey-Kramer post-test were used to investigate the statistical difference. #### *P*<0.0001 in comparison to the control group, **** *P*<0.0001, *** *P*<0.001, * *P*<0.05 compared to the ACR group.

ACR: Acrylamide; Sita: Sitagliptin; Vit E: Vitamin E

ACR (50 mg/kg), sitagliptin (5, 10, 20, and 40 mg/kg), and vitamin E (200 mg/kg) were injected intraperitoneally into the animals. The data is Mean±SD (n=7). ANOVA test and Tukey-Kramer post-test were used to investigate the statistical difference. ### *P*<0.001 versus the control group and *** *P*<0.001 versus the ACR group.

ACR: Acrylamide; Sita: Sitagliptin; Vit E: Vitamin E

A: Specific bands related to the level of TNF-α, which were examined by western blotting. B: Data on the TNF-α level results from the densitometric analysis. The data is Mean±SD (n=4). ANOVA test and Tukey-Kramer post-test were used to investigate the statistical difference. ##* P*<0.01 compared with the control group and * *P*<0.05 compared to the ACR receiving group.

ACR: Acrylamide; Sita: Sitagliptin; Vit E: Vitamin E

A: Specific bands related to the level of IL-1β, were examined by western blotting. B: Data on the TNF-α level results from the densitometric analysis. The data is Mean±SD (n=4). ANOVA test and Tukey-Kramer post-test were used to investigate the statistical difference. ## *P*<0.01 compared to the control group.

ACR: Acrylamide; Sita: Sitagliptin; Vit E: Vitamin E

A: Specific bands related to caspase-3 proteins (pro & cleaved) that have been investigated by western blotting. B: Data on the level of caspase-3 proteins (pro & cleaved) are the result of the densitometric analysis. The data is Mean±SD (n=4). ANOVA test and Tukey-Kramer post-test were used to investigate the statistical difference. ## *P*<0.01 compared to the control group and * *P*<0.05 compared to the ACR group.

ACR: Acrylamide, Sita: Sitagliptin; Vit E: Vitamin E

## Discussion

The current research examined the potential therapeutic and protective properties of sitagliptin against neurotoxicity induced by ACR in rats. The present results indicate that the injection of ACR (50 mg/kg) for 11 days significantly led to movement disorders, decreased GSH content, and increased amounts of MDA, TNF-α, IL-1β, and caspase-3, resulting in the induction of oxidative stress, inflammation, and apoptosis in the brain cortex. Supplementation of sitagliptin (5, 10, 20, and 40 mg/kg, 11 days) revealed that the most efficient dose was 10 mg/kg, which reversed the ACR-induced changes but had no significant effect on IL-1β. Sitagliptin (10 mg/kg) in all three protocols (from the first day to day 11, from day 6 to 11, and day 6 to 20) was able to reduce ACR movement disorders. Co-administration of sitagliptin (5, 10, 20, and 40 mg/kg) concomitantly with ACR for 11 days augmented GSH levels in brain tissues in rats. Injection of sitagliptin (5, 10, and 20 mg/kg) with ACR, as well as administration of sitagliptin 10 mg/kg from day 6 with ACR for 11 days, lessened MDA amounts in brain tissue, and receiving vitamin E with ACR, which was as a positive control group, reduced movement disorders, decreased MDA, increased GSH content, reduced TNF-α and caspase-3 levels in cortex tissue compared to the ACR receiving group. 

ACR is a human neurotoxin that is formed by the cooking of starchy foodstuffs at temperatures exceeding 120 °C, such as breakfast cereals, bread, chips, and coffee (1, 2). It causes damage to the central and peripheral nervous systems (4). The most common mechanisms underlying ACR-induced neurotoxicity are the production of oxidative stress in neurons, induction of apoptosis (2), and triggering of neuroinflammation (12).

Exposure to ACR has also been found to harm cellular macromolecules and degrade neurons, which are the primary roots of motor dysfunction (34). Neuropathies, numbness in the arms and legs, irregular gait, muscle weakness, and ataxia are all symptoms of chronic exposure to the toxin (11). Mehri *et al*. observed that intraperitoneal injection of 50 mg/kg ACR to male Wistar rats for 11 days, caused severe movement disorders (35). Also, it has been shown that intraperitoneal administration of ACR (50 mg/kg, 11 days) to male Wistar rats caused movement disorders (36). In the present study, intraperitoneal injection of ACR at a dose of 50 mg/kg for 11 days significantly increased movement disorders, increased gait scores and hind limb paralysis in rats. In contrast, co-administration of sitagliptin (10 mg/kg) in all three protocols (from the first day to day 11, from day 6 to 11, and day 6 to 20) was able to reduce ACR movement disorders and lessen paralysis. Other doses of sitagliptin (5, 20, and 40 mg/kg) also amended ACR-induced movement disorders, nonetheless it was not significant. 

Moreover, previous investigations have indicated that intraperitoneal injection of ACR (50 mg/kg, 11 days) significantly lowered GSH levels and raised MDA levels (22, 37). In line with previous studies, the present research data showed that ACR (50 mg/kg) pointedly augmented MDA levels and declined GSH content. In our study, co-administration of sitagliptin (5, 10, 20, and 40 mg/kg) concomitantly with ACR for 11 days augmented GSH levels in brain tissues in animals that received ACR. Supplementation of sitagliptin (5, 10, and 20 mg/kg) with ACR, as well as administration of sitagliptin 10 mg/kg from day 6 with ACR for 11 days decreased MDA levels in brain tissue. Previous studies showed the anti-oxidant effect of sitagliptin. In kainic acid-induced status epilepticus in rats, sitagliptin (5 and 10 mg/kg, IP) protected against oxidative stress through increasing SOD besides decreasing MDA amount (38).

Many neurological illnesses are characterized by central nervous system inflammation. Microglia, which are intrinsic immune cells located in the brain, have a crucial function in monitoring the immune system and clearing cellular debris. Activated microglia produce IL-1β and other pro-inflammatory cytokines comprising TNF-α and IL-6, which lead to inflammation in the brain and nerve damage (39). ACR has been found to promote inflammation and cell damage by up-regulating the expression of genes involved in the generation of inflammatory factors including TNF-α and IL-1 β (40). A study has shown that ACR (40 mg/kg/day, 4 weeks) in rats can increase the inflammatory cytokines TNF-α and IL-1β in brain tissues (41). Another study reported that oral administration of ACR (20 mg/kg, 30 days) increased TNF-α and IL-1β levels in brain tissue (13). Our findings showed that ACR (50 mg/kg, 11 days) increased the level of TNF-α and IL-1β in cortical tissue versus the control group. However, administration of sitagliptin (10 mg/kg) with ACR for 11 days was able to significantly reduce the level of TNF-α protein in the cerebral cortex versus the ACR group. Sitagliptin administration reduced IL-1β protein levels versus the ACR group although it was not significant. Consistent with our results, sitagliptin (600 mg/kg/day, 90 days, PO) reduced cognitive impairment and brain damage in rats suffering from chronic cerebral hypo-perfusion by decreasing inflammation via attenuating IL-1β, TNF-α, and other inflammatory factors (42). 

Exposure to cytokines has been shown to prompt apoptosis in neurons (43). The Bcl-2 family is made up of about twenty proteins that work together to keep cells alive or trigger apoptosis (44). It contains multiple Bcl2-related genes, some of which increase apoptosis (such as Bax), while others inhibit apoptosis (including Bcl-2) (45). The important indicator of apoptosis is the activation of caspases (46). During apoptosis, many caspases are activated; for example, caspase-3 is a downstream effector that plays a key role in the start of apoptosis (47). Apoptosis is another important mechanism in ACR neurotoxicity. In a study, ACR administration (50 mg/kg, 14 days, PO) amplified the levels of caspase-3 in rat cortex samples (48). Besides, ACR (50 mg/kg, 11 days, IP) augmented the levels of caspase-3 in the cortical tissue of the brain and liver (6). In our research, following the injection of ACR (50 mg/kg, 11 days, IP) there was no substantial variation in the level of procaspase-3 protein in cortical tissue. ACR administration also significantly increased the level of caspase-3 cleaved protein in cortical tissue. On the other hand, administration of sitagliptin at a dose of 10 mg/kg with ACR pointedly attenuated the level of cleaved caspase-3 protein in the cortex of the brain versus the ACR group. In previous studies, sitagliptin exhibited neuroprotective effects through anti-apoptotic properties. It was reported that treatment with sitagliptin (250 mg/kg, 2 weeks, PO) significantly revealed an anti-apoptotic effect by reducing caspase-3 amount in the hippocampus of diabetic rats with cerebral ischemia/reperfusion injury (19).

In this study, vitamin E was employed as a positive control, supplementing vitamin E with ACR reduced movement disorders, reduced MDA, increased GSH content, and significantly reduced TNF-α, and caspase-3 levels in cortex tissue compared to the ACR receiving group. The protective effects of vitamin E on ACR-induced toxicity have been demonstrated in several studies. For example, vitamin E (200 mg/kg) administered intraperitoneally lessened the neurotoxicity generated by 50 mg/kg ACR on brain tissue by lowering MDA levels, increasing GSH content, and lowering cleaved caspase-3 levels (25).

## Conclusion

Intraperitoneal administration of ACR at a dose of 50 mg/kg for 11 days causes movement disorders in the tested rats and prompts oxidative stress, inflammation, and apoptosis in the cortical tissue of the brain. Co-administration of sitagliptin with ACR reduces rat movement disorders, indicating the therapeutic and protective effects of sitagliptin on ACR-induced toxicity. Sitagliptin is also effective in protecting against ACR-induced neurotoxicity through its anti-oxidant, anti-inflammatory, and anti-apoptotic effects. According to the results, it can be concluded that sitagliptin can have prophylactic and therapeutic effects against ACR toxicity and can be used as an efficacious combination in the prevention and treatment of neurotoxicity. Of course, to better understand the mechanisms of its effect, more studies are needed in this field.

## Authors’ Contributions

H H and S M supervised the research and designed the experiments; M N performed the experiments; M GR supervised the implementation of the experiments and wrote the manuscript; all authors reviewed the results and approved the final version of the manuscript.

## Conflicts of Interest

The authors declare that they have no conflicts of interest.
